# Gel Polymer Electrolytes with Mixture of Triazolium Ionic Liquids and Propylene Carbonate

**DOI:** 10.3390/gels8060370

**Published:** 2022-06-12

**Authors:** Aneta Lewandowska, Piotr Gajewski, Katarzyna Szcześniak, Agnieszka Marcinkowska

**Affiliations:** Institute of Chemical Technology and Engineering, Poznan University of Technology, Berdychowo 4, 60-965 Poznan, Poland; piotr.gajewski@put.poznan.pl (P.G.); agnieszka.marcinkowska@put.poznan.pl (A.M.)

**Keywords:** triazolium ionic liquids, gel polymer electrolytes, thiol–ene polymerization, Kamlet–Taft parameters

## Abstract

This study is focused on the structural influence of 1,2,4-triazolium ionic liquid (IL), that is, the effect of the length of the substituent and the type of substitution (1-methyl-4-alkyl or 1-alkyl-4-methyl) used in the mixture with propylene carbonate (PC) on the properties of thiol–ene polymer ionogels and on the preparation of an ionogel with satisfactory mechanical and conductive properties. PC allows for higher conductivity but also causes electrolyte leakage from the gel. When using triazolium IL (instead of the imidazolium one), because of the stronger interactions between components of the system, the ionogels do not leak. In this study, 1,4-dialkyl-1,2,4-triazolium ILs were successfully synthesized by the alkylation of 1,2,4-triazole. Subsequently, gel polymer electrolytes were obtained by one-pot thiol–ene photopolymerization reactions of tetrafunctional thiols with different chemical structures: pentaerythritol tetra(3-mercaptopropionate) (PETMP) or pentaerythritol tetra(3-mercaptobutyrate) (PETMB) and trifunctional ene (TATT) in the presence of a mixture of 1,4-dialkyl-1,2,4-triazolium IL with PC. Measurements made by electrochemical impedance spectroscopy showed that all ionogels with TATT+PETMB as a polymer matrix presented smaller relative ionic conductivity compared to ionogels containing TATT+PETMP. The puncture resistance and elongation at puncture, measured by the puncture resistance method, were higher for ionogels with poly(TATT+PETMB) than for those with poly(TATT+PETMP). Moreover, ILs containing a methyl group in position N1 of the 1,2,4-triazole ring presented lower puncture resistance than ionogels with ILs containing a methyl group in position N4, especially for shorter alkyl chains. Additionally, the photo-differential scanning calorimetry method was employed to characterize the course of photopolymerization. The compositions and their constituents were characterized by UV and IR spectroscopy.

## 1. Introduction

Liquid electrolytes have played a fundamental role in electrochemical energy storage devices for many years thanks to their excellent contact with materials used as electrodes, as well as ionic conductivity at the level of about 10^−2^ S·cm^−1^ [1]. Unfortunately, the use of liquid electrolytes also entails the risk of electrolyte leakage and the ignition of organic electrolytes, resulting in problems with the waste disposal and management of these flammable liquid solvents, in addition to the unavoidable increase in lithium dendrite growth in liquid solution (caused by uneven currents) [2]. This is why, taking into account all of the above-mentioned issues, and to maintain all of the requirements for electrolytes needed for conventional batteries such as, firstly, environmental sustainability and low flammability, secondly, electrochemical as well as chemical stability towards the electrodes, and, most importantly, high conductivity, another approach is needed [3]. Moreover, taking into consideration extensive increases in the automotive industry and the process of bringing new lithium-ion batteries to the market—which have grown a lot and found a wide variety of applications such as in electric vehicles, cell phones, and other devices—the next generation, a new type of electrolyte, needs to be created by taking into account the most important features of the sustainable development of ecological factory-made processes, i.e., looking for novel materials with lowered or no toxicity, and taking into consideration problems associated with the disposal, recycling, and reuse of batteries [4].

In recent years, several new concepts have been proposed to design and obtain new quasi-solids and solid electrolytes with increased safety properties, permanence and electrochemical performance. One of the promising approaches is the usage of solid polymer electrolytes (SPEs) and gel polymer electrolytes (GPEs). Both of these types of electrolytes are broadly studied in lithium-ion batteries. SPEs are characterized by excellent electrochemical performance, high safety, and good quality. Moreover, properties such as good flexibility and process capability are also very important and will have great applicability in new-generation high-energy batteries [5,6,7]. Many different systematic investigations of SPE materials have been carried out in order to establish an effective balance of SPEs between two phases (crystalline and amorphous) and to come to new conclusions in terms of ionic conductivity and mechanical properties, including the synthesis of a new polymer matrix, e.g., with polyethylene oxide (PEO) [8], polycarbonate [9], and polysiloxane [9,10]. Unfortunately, despite many advantages, the mechanical strength of SPEs is still not as good as it should be, which is the main obstacle hindering their broad application, as well as their low conductivity of around *σ* = 10^−6^ S·cm^−1^ at numerous temperatures. This is mostly because a crystalline phase is present in the polymer, which limits their extensive application.

In turn, GPEs comprise a mixture of liquid electrolytes and solid polymer formulations in such a way as to obtain a new material with the mechanical properties of solid polymers and the ionic conductivity of liquid electrolytes. This kind of approach ensures a flexible, fluid-filled (in its interstitial space), cross-linked polymer network. Thus, the network can limit the location of the fluid in a specific space, while the gel maintains the elasticity of the fluid shape. Comparing SPEs with GPEs, the second one is characterized by greater ionic conductivity at ambient temperature [11]. In GPEs, the liquid electrolyte is trapped in a polymer matrix, and the risk of leakage can be reduced in comparison to the commercially available separator. A huge variety of polymers can be found on the market today; therefore, many different GPEs have been researched and described in the literature. The majority of GPEs are based on poly(methyl methacrylate), very broadly used polymers such as poly(ethylene oxide) and polyacrylonitrile, and mainly on poly(vinylidene fluoride) homo- and copolymers [12]. The most popular ways to prepare GPEs are the chemical polymerization of monomers, the physical gelation of polymers, and the crosslinking of polymers in the presence of liquid electrolytes. Due to recent improvements that have been achieved thanks to composition modifications and the introduction of various additives (i.e., organic and inorganic nanofillers), ionic liquids are willingly and often described. Interesting possibilities are provided by ionic liquids that are inherently non-flammable [13] and therefore safer. In a significant way, they can improve cyclicity as pure liquids [14,15], or they can be embedded in polymer matrices to create GPEs. Recently, more and more research has focused on ionic liquids (IL) due to their suitability as new and “greener” solvents [16], as their ability to be useful as a new solvent medium is quite sensational. ILs provide the possibility of new catalytic behavior, thanks to their very interesting and useful properties, including non-existent vapor pressures, high relative permittivity, high electrical conductivity, and frequently outstanding thermal and—even more importantly—chemical stability [17]. Although the 1,3-dialkylimidazolium cation occupies the most superior position in the field of ionic liquids, there is still a strong need to learn about ionic liquids from other groups with the aim of adapting their physical properties to a particular purpose [13,18]. While the interest in ionic liquids containing 1,2,4-triazole has increased over the past ten years, most research has focused on materials specialized in the energy sector [19]. Ionic liquids with a triazole nitrogen-rich ring, compared to an imidazole ring, show quite substantial positive heats of formation, ameliorated thermal stabilities, enhanced densities, and diminished vapor tension [20].

However, gel electrolytes still suffer from instability over time, both as a result of washing the liquid from the membrane and as a result of chemical and electrochemical reactions at the electrode/electrolyte interface. This is why here in this paper we want to present, firstly, the synthesis of a 1,2,4-triazolium-containing ionic liquids and, later, the synthesis of ionogels containing instead of pure ILs, the mixture of ILs with propylene carbonate, to reduce the costs of the ionogels and to increase their ionic conductivity. Previously we reported the successful synthesis of GPEs using the thiol–ene reaction [21,22]. This method of synthesizing GPE allows for the production of tailorable materials [23] and combines the classic advantages of the click reaction with the advantages of the process initiated by UV-light. Comparing the photopolymerization of (meth)acrylate to the synthesis using thiol, it is essential to notice that no oxygen inhibition is observed, which makes the process (UV curing) independent of the presence of oxygen and also delays the gel point conversion. All these observed properties, as well as a reduction in the shrinkage stress generated during polymerization, causes the formation of a polymer network with narrow glass transition regions, which is ideally homogeneous [24].

## 2. Materials

### 2.1. Reagents for Ionic Liquid Synthesis

1,2,4-triazole, 98%; iodomethane (IM, 99%); iodoethane (IE, 99%); 1-iodopropane (IP, 99%); 1-iodobutane (IB, 99%); bromoethane (BE, 98%); 1-bromopropane (BP, 99%); 1-bromobutane (BB, 99%); bis(trifluoromethane)sulfonimide lithium salt, 99%; and acetonitrile obtained from Sigma-Aldrich (St. Louis, MO, USA).

### 2.2. Other Reagents

Pentaerythritol tetra(3-mercaptobutyrate) (PETMB, 97%) generously given by Showa Denko KK (Tokyo, Japan); 1,3,5-tri-2-propenyl-1,3,5-triazine-2,4,6 (1H,3H,5H)-trione (TATT, 98%), pentaerythritol tetra(3-mercaptopropionate) (PETMP, 95%), benzil α,α-dimethyl acetal (DMPA), and propylene carbonate (PC, anhydrous, 99.7%) bought from Sigma–Aldrich.

## 3. Methodology

### 3.1. Synthesis of 1,4-Dialkyl-triazolium Ionic Liquid

**Figure 1:**
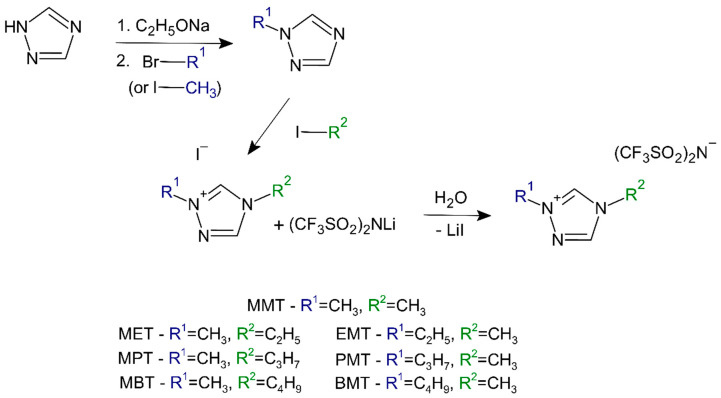
Synthesis of ionic liquids.

1-alkyl-1,2,4-triazoles were prepared by the alkylation of 1,2,4-triazole with 1-bromoalkanes (BE, BP, BB) or iodomethane. At first, the dissolution of one mole of 1,2,4-triazole was performed in sodium ethoxide (one mole of sodium was added to ethanol to obtain a solution of sodium ethoxide in ethanol). Then, 1 mole of IM, BE, BP, and BB was added and heated to the reflux temperature of ethanol for 2 h. When the reaction was finished, the separated sodium bromide or iodide was filtered off, and the ethanol was evaporated. The respective 1-alkyl-1,2,4-triazoles were distilled under reduced pressure to obtain 1-methyl-1,2,4-triazole, 1-ethyl-1,2,4-triazole, 1-propyl-1,2,4-triazole, or 1-butyl-1,2,4-triazole. Regioselective N-1 alkylation of 1,2,4-triazole generally takes place at the N-1 position and not at the N-4 position of the triazole ring. However, it is possible to obtain small amounts of the N-4 product in the case of the methylation reaction. These undesirable reaction products must be separated under vacuum distillation. 1,2,4-triazoles were functionalized in the N-1 position by reacting first with sodium ethoxide and then with the addition of the appropriate bromoalkenes or iodomethane [25]. In the next step, the quaternization of the obtained 1-alkyl-1,2,4-triazoles was performed in the N-4 position using appropriate alkyl iodides. Then, 0.1 moles of the resulting compounds was quaternized in acetonitrile with 0.11 moles of IM, IE, IP, or IB, respectively. The 1,4-dialkyl-1,2,4-triazolium iodides thus obtained were purified on activated carbon. For pure compounds, anion exchange reactions were performed, in which the iodine anion was exchanged to trifluoromethylsulfonyl)imides (Figure 1). All synthesized 1,4-dialkyl-1,2,4-triazolium bis(trifluoromethylsulfonyl)imide ionic liquids, together with their acronyms, are shown in Table 1.

On the basis of nuclear magnetic resonance, ^13^C NMR and ^1^H NMR spectra of the synthesized triazolium ionic liquids were determined. NMR spectra were recorded using a Bruker spectrometer (internal standard: tetramethylsilane) at 100 MHz and 400 MHz for ^13^C NMR and ^1^H NMR, respectively.

The NMR measurement results are as follows:

1,4-dimethyl-1,2,4-triazolium bis(trifluoromethylsulfonyl)imide MMT: ^1^H NMR (400 MHz, DMSO-d6) δ ppm: 3.89 (s, 1H); 4.06 (s, 1H); 9.09 (s, 1H); 9.97 (s, 1H). ^13^C NMR (100 MHz, DMSO-d6) δ ppm: 33.95; 38.52; 114.72; 117.92; 121.12; 124.32; 143.38; 145.36.

4-ethyl-1-methyl-1,2,4-triazolium bis(trifluoromethylsulfonyl)imide MET: ^1^H NMR (400 MHz, DMSO-d6) δ ppm: 1.44–1.48 (t, 3H, *J* = 7.3 Hz); 4.06 (s, 3H); 4.25–4.30 (q, 2H, *J* = 7.3 Hz); 9.20 (s, 1H); 10.07 (s, 1H). ^13^C NMR (100 MHz, DMSO-d6) δ ppm: 14.49; 38.52; 42.99; 114.72; 117.91; 121.12; 124.32; 142.70; 144.32.

1-methyl-4-propyl-1,2,4-triazolium bis(trifluoromethylsulfonyl)imide MPT: ^1^H NMR (400 MHz, DMSO-d6) δ ppm: 0.89–0.93 (t, 3H, *J* = 7.4 Hz); 1.81–1.90 (m, 2H); 4.07 (s,3H); 4.20–4.23 (t, 2H, *J* = 7.2 Hz); 9.19 (s, 1H); 10.07 (s, 1H). ^13^C NMR (100 MHz, DMSO-d6) δ ppm:10.30; 22.44; 38.62; 48.92; 114.70; 117.90; 121.10; 124.30; 142.84; 144.48.

4-butyl-1-methyl-1,2,4-triazolium bis(trifluoromethylsulfonyl)imide MBT: ^1^H NMR (400 MHz, DMSO-d6) δ ppm: 0.90–0.94 (t, 3H, *J* = 7.4 Hz); 1.27–1.36 (m, 2H); 1.77–1.85 (m, 2H); 4.06 (s, 3H); 4.23–4.27 (t, 2H, *J* = 7.3 Hz); 9.20 (s, 1H); 10.07 (s,1H). ^13^C NMR (100 MHz, DMSO-d6) δ ppm:13.18; 18.73; 30.88; 38.62; 47.17; 114,69; 117.89; 121.09; 124.29; 142.83; 144.74.

1-ethyl-4-methyl-1,2,4-triazolium bis(trifluoromethylsulfonyl)imide EMT: ^1^H NMR (400 MHz, DMSO-d6) δ ppm: 1.46–1.48 (t, 3H, *J* = 7.3 Hz); 3.89 (s, 3H); 4.37–4.42 (q, 2H, *J* = 7.3 Hz); 9.10 (s, 1H); 10.03 (s, 1H). ^13^C NMR (100 MHz, DMSO-d6) δ ppm: 13.65; 33.91; 47.02; 114.71; 117.91; 121.11; 124.31; 142.65; 145.39.

4-methyl-1-propyl-1,2,4-triazolium bis(trifluoromethylsulfonyl)imide PMT: ^1^H NMR (400 MHz, DMSO-d6) δ ppm: 0.88–0.92 (t, 3H, *J* = 7.4 Hz); 1.83–1.92 (m, 2H); 3.90 (s, 3H); 4.32–4.35 (t, 2H, *J* = 7.0 Hz); 9.12 (s, 1H); 10.04 (s, 1H). ^13^C NMR (100 MHz, DMSO-d6) δ ppm: 10.03; 21.71; 33.98; 53.02; 114.72; 117.91; 121.12; 124.32; 143.01; 145.51.

1-butyl-4-methyl-1,2,4-triazolium bis(trifluoromethylsulfonyl)imide BMT: ^1^H NMR (400 MHz, DMSO-d6) δ ppm: 0.86–0.90 (t, 3H, *J* = 7.4 Hz); 1.26–1.31 (m,2H); 1.79–1.83 (m, 2H); 3.87 (s, 3H); 4.31–4.35 (t, 2H, *J* = 7.0 Hz); 9.05 (s, 1H); 10.02 (s, 1H). ^13^C NMR (100 MHz, DMSO-d6) δ ppm: 13.08; 18.73; 30.19; 33.97; 51.36; 114.79; 117.99; 121.19; 124.38; 143.05; 145.50.

### 3.2. Solvatochromic Parameters

Solvatochromic parameters were determined for monomers and mixtures of triazolium IL with PC. Using appropriate reagents, i.e., 4-nitroaniline, (N, >99%), *N*,*N*-diethyl-4-nitroaniline (DN, 98%), and Reichardt’s dye (RD, 90%), all delivered by Sigma-Aldrich the empirical Kamlet–Taft polarity parameters (hydrogen bond donating ability—*α*; hydrogen bond accepting ability—*β*; dipolarity/polarizability—π*), as well as Reichardt’s empirical polarity parameters ($E_{T}$(30) and normalized$\;E_{T}^{N}$ were determined. Solvatochromic parameters were estimated according to the method described previously [26] using spectrophotometer Jasco UV-530 (Tokyo, Japan) and calculated according to Equations (1)–(5) and methods presented in the references [27,28,29,30]:

(1) $$\alpha =\frac{E_{T}\left(30\right)-14.6\left(\pi^{*}-0.23\right)-30.31}{16.5}$$

(2) $$\beta =\frac{1.035v{\left(DN\right)}_{max}-v{\left(N\right)}_{max}+2.64}{2.8}$$

(3) $$\pi^{*}=\frac{v{\left(DN\right)}_{max}-27.52}{-3.182}$$

(4) $$E_{T}\left(30\right)=\frac{28591}{\lambda {\left(RD\right)}_{max}}$$

(5) $$E_{T}^{N}=\frac{E_{T}\left(30\right)-30.7}{32.4}$$

in which DN and N are nitroaniline reagents *N*,*N*-diethyl-4-nitroaniline and 4-nitroaniline, respectively, and RD is Reichardt’s dye.

### 3.3. Isothermal Differential Scanning Photocalorimetry

Isothermal differential scanning calorimetry was used for the kinetics studies of photopolymerization of the compositions with and without solvents. Measurements were made on a DSC apparatus (DSC, Pyris 6 instrument, Perkin Elmer, Waltham, MS, USA) equipped with an accessory for photopolymerization studies. Samples of 1.7 ± 0.1 mg of the studied mixtures were photocured at 25 °C (isothermal conditions). The crucibles, with a diameter of 6.6 mm, were open so that UV light (LED lamp, Hamamatsu Photonics, Hamamatsu Japan LC-1, *λ* = 365 nm, *I*_0_ = 1 mW·cm^−2^) directly irradiated the sample. Polymerization was carried out in an inert gas atmosphere (Ar with high-purity, <0.0005% of O_2_); that is, the sample was purged with a gas flowing at a rate of 20 mL·min^−1^ both one minute before and during the polymerization reaction. All thiol–ene reactions were performed at least three times (the results were nearly 3% repeatable).

### 3.4. Infrared Spectroscopy

Infrared (IR) spectra of the pristine triazolium ILs, thiol monomers, as well as mixtures of reagents used (thiol–ene, thiol–ene-IL-PC) were carried out on an infrared Nexus Nicolet 5700 (FTIR, Thermo Electron Scientific Instruments Corporation, Madison, WI, USA). The FTIR apparatus was equipped with an accessory for measurements in attenuated total reflection. The measurements conditions were as follows: room temperature, diamond crystal, resolution 4 cm^−1^ at 64 scans, range 4000–600 cm^−1^. Equation (6) was used to determine infrared spectra shifts (Δν) of the appropriate absorption bands: the C3-H and C5-H of the triazole ring of the synthesized ILs and the SH group of the thiols:

(6) $$\Delta \nu =\nu_{c}-\nu_{0}$$

in which Δν is the difference between the absorption peak location of the selected group (C3-H, C5-H, SH) of the studied compound in the mixture ($\nu_{c}$) and in the pure compound ($\nu_{0}$).

### 3.5. Gel Polymer Electrolyte Synthesis

**Figure 2:**
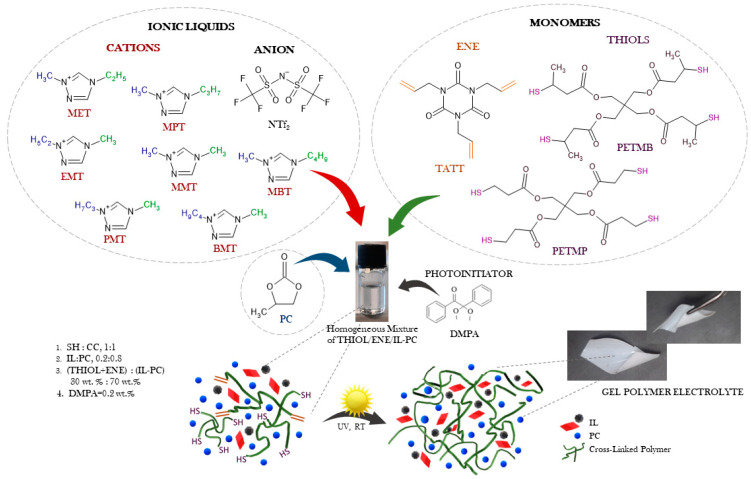
Schematic illustration of GPEs synthesis.

The preparation of gel polymer electrolytes by one-pot reaction of primary or secondary tetrafunctional thiol (PETMP, PETMB) with trifunctional ene (Sigma-Aldrich) in the presence of a mixture of ionic liquid with PC is shown in Figure 2. The ratio of the functional thiol group (SH) to the monomer ene group (C=C) in the mixture (thiol–ene) was stoichiometric (1:1). The solvent mixture (0.8 mol:0.2 mol, PC:IL) was used in the compositions studied at a concentration of 70 wt.%. The concentration of DMPA (photoinitiator) was calculated based on the entire composition—0.2 wt.%. The photocurable compositions were prepared under an inert gas atmosphere (Ar) in a glovebox. The homogeneous mixtures of monomers (thiol, ene), solvents (IL, PC), and photoinitiator were prepared in an orbital shaker, and immediately after mixing, they were poured into a mold consisting of two glass plates with 0.3 mm thickness between them. The samples were exposed to UV light (ASN-36W UV lamp, *λ_max_* = 365 nm, *I*_0_ = 6 mW·cm^−2^) at room temperature on both sides of the mold for 5 min in the case of the compositions containing primary thiol PETMP or 10 min for the compositions with secondary thiol PETMB. Then, from the sheets of the obtained ionogels, the specimens of suitable dimensions were cut.

### 3.6. Puncture Resistance

Puncture resistance tests on the CT3 texture analyzer (Ametek Brookfield, Middleboro, MA, USA) were selected for studies of the mechanical properties of prepared ionogels. These studies were carried out according to the methodology described in the previous work [26]. Briefly, a sample (16 mm) was mounted in a holder (hole 10 mm) and tested for puncture resistance with the probe (cylindrical, Ø 5 mm, displacement rate 0.3 mm·s^−1^). Distance and probe load were collected during measurements until material perforation. Additionally, load normalization to a uniform thickness of the ionogels (300 µm) was performed. Each ionogel was measured five times, and the average values and standard deviations were calculated.

### 3.7. Differential Scanning Calorimetry

Differential scanning calorimetry was used to determine the glass transition temperatures of ionogels T_g_ (DSC1 apparatus Mettler-Toledo, Greifensee, Switzerland). The rate of heating/cooling was equal to 20 °C·min^−1^, and measurements were performed under an inert gas atmosphere (argon, 50 mL·min^−1^). The temperature range was established from −80 °C to 100 °C. For the determination of glass transition temperatures T_g_, the second heating cycle was taken.

### 3.8. Electrochemical Impedance Spectroscopy

The electrochemical impedance spectroscopy (EIS) with SP-300 potentiostat/galvanostat (Biologic, Seyssinet-Pariset, France) was used to determine the ionic conductivity of ILs, IL + PC mixtures, and GPEs. Measurements were carried out in the 1 kHz–1 MHz frequency range, at room temperature. The measurement of ionic conductivity of liquid electrolytes was performed in dedicated electrochemical vessel with two platinum electrodes (constant of the vessel K = 1.54 cm^−1^). For ionic conductivity measurement of ionogels, a self-designed vessel was applied (Swagelok-type vessel where electrodes–stainless steel 316 L—were directly connected with micrometer to measure the thickness of the sample).

The ionic conductivity of mixtures of ionic liquids with PC ($\sigma_{IL,PC}$) was calculated from Equation (7):

(7) $$\sigma_{IL,PC}=K\cdot \sigma_{s}$$

where *K* is the constant of the vessel (cm^−1^), and $\sigma_{s}$ is the volumetric conductance of the ionic liquid in PC (S).

Moreover, Equation (8) was used to calculate the ionic conductivity of the GPEs (*σ*):

(8) $$\sigma =\frac{d\cdot \sigma_{s}}{A}$$

in which *d* is thickness (cm), $\sigma_{s}$ is volumetric conductance (*S*), and *A* is the ionogel surface area (cm^2^) of the GPE.

Volumetric conductance of the ionic liquid in PC and ionogels was obtained as the plateau at the high-frequency region on the conductance vs. frequency plot.

To compare the conductivity of ionogels containing ILs with different conductivity values, the relative conductivity $\sigma_{rel}$ of prepared GPEs in relation to the $\sigma_{IL,PC}$ was calculated (Equation (9)):

(9) $$\sigma_{rel}=\frac{\sigma }{\sigma_{IL,PC}}\;100\%$$

### 3.9. Electrochemical Capacitor Investigation

#### 3.9.1. Electrodes Preparation

The activated carbon (AC) electrodes were prepared by mixing the 85 wt.% of AC (Maxsorb MSP-20X, Kansai Coke and Chemicals CO, Hyogo, Japan) with carbon black (CB)–10 wt.% (C-NERGY™ SUPER C65, Imerys S. A., Paris, France) and binder–10 wt.% (60 wt.% water dispersion of PTFE, Sigma–Aldrich). The components were mixed together in deionized water. Then, the water was partially evaporated and the thin electrode film was prepared by rolling-out to an average thickness of 150 ± 12 μm. Thereafter, electrodes were dried in vacuum oven and later glued to the etched stainless steel current collector (316L) using Acheson electrodag (PF-407C, Henkel, Dusseldorf, Germany). The electrodes with the average weight of 50 ± 4 mg and size of 20 mm × 25 mm and the thin film (d = 200 ± 15 μm) of gel polymer electrolyte with the size of 25 mm × 30 mm were applied in a pouch-type symmetric AC/AC capacitor.

#### 3.9.2. Electrochemical Investigation

The electrochemical double-layer capacitor (EDLC) with one of the synthesized gels was evaluated in a two-electrode symmetrical AC/AC pouch-cell. Before assembling, the carbon electrodes were soaked off in a solution of IL with PC, and assembled in the argon atmosphere with the gel polymer electrolyte as a separator. The cell was investigated by using cyclic voltammetry (CV) with the scan rate from 2 to 100 mV·s^−1^ and up to maximal cell potentials of 1–3.0 V, as well as galvanostatic charge/discharge with potential limitation (GCPL, 0.2–3 A·g^−1^) and electrochemical impedance spectroscopy (EIS) at open-circuit voltage (OCV, frequency range from 0.5 MHz to 1 mHz with a 10 mV amplitude) by using an SP300 potentiostat/galvanostat (Biologic, Seyssinet-Pariset, France).

### 3.10. Scanning Electron Microscope

The scanning electron microscope JEOL 7001F (SEI detector, 15 kV accelerating voltage) was used for morphology studies of the GPEs obtained. Before measurements, mixtures of ILs with PC were washed out of the samples with methanol. Next, to remove methanol from the polymer matrices, they were dried in an oven (temperature 35 °C). After that, the small piece of prepared polymer matrix was placed on a stub of metal with adhesive. The ultrathin Gold/Palladium layer was then deposited on the sample by low-vacuum sputter coating.

## 4. Results and Discussion

### 4.1. Characterization of Components and Compositions

**Figure 3:**
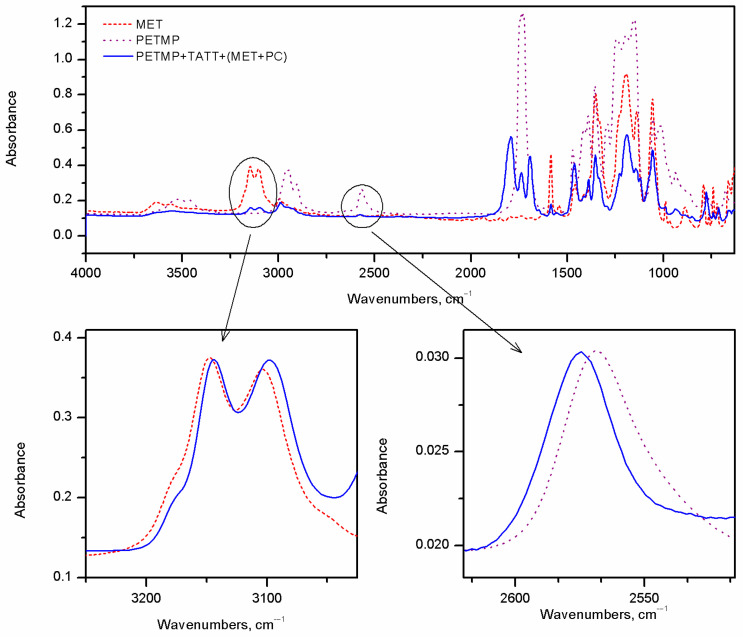
FTIR spectra of pristine PETMP (thiol monomer), METNTf_2_ (triazolium ionic liquid), and a composition of PETMP+TATT+MET+PC. Additionally, shifts in the investigated absorption bands C3-H and C5-H of the triazole ring and SH group of thiol are shown (the absorbance has been normalized to show shifts in the studied bands).

**Figure 4:**
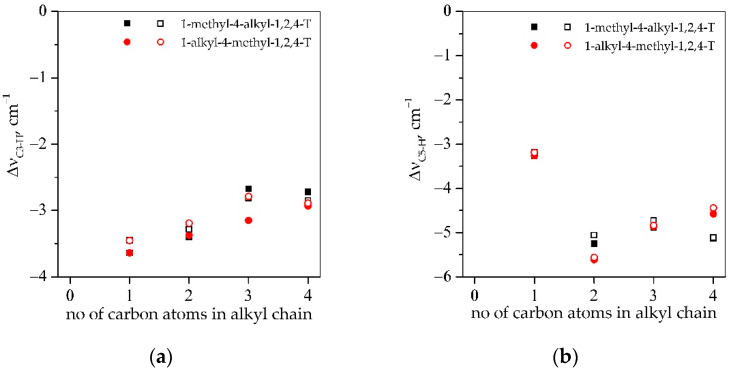

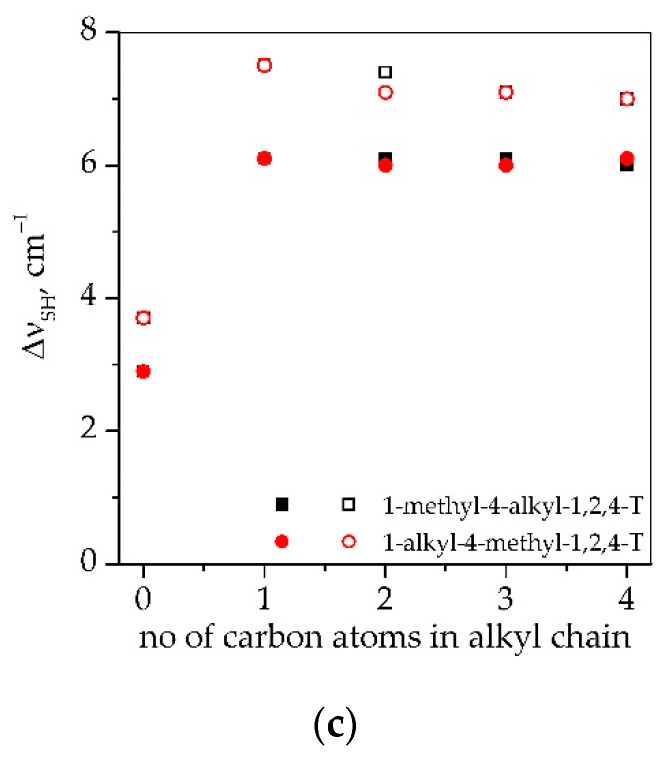
Results of FTIR investigations: shifts in the absorption bands of the (**a**) C3-H *(*$∆\nu_{C3-H}$) and (**b**) C5-H ($∆\nu_{C5-H}$) bonds of 1,2,4-triazole ring of the ionic liquids and (**c**) SH ($∆\nu_{SH}$) group of the thiols PETMP (filled symbols) and PETMB (open symbols) in four-component systems (thiol–ene–IL–PC).

Infrared spectroscopy was used to investigate the intermolecular interactions between components of the studied compositions. The interactions between used reagents were studied for thiol–ene systems, i.e., PETMB+TATT and PETMP+TATT, in two-component mixtures (thiol with ene), as well as three-component (thiol with ene and solvent) or rather four-component mixtures, because the solvent was a mixture of triazolium ionic liquid and propylene carbonate. The position of the absorption band of the thiol SH group, as well as the C3-H and the C5-H of the triazole ring, was observed. The shifts in these absorption bands in the mixtures of monomers with or without solvents with reference to the pristine compounds, i.e., thiol and ionic liquid, are shown in Figure 3 and Figure 4. As can be found in the literature [31,32,33], mercaptopropionates are capable of forming hydrogen bonds between the hydrogen of an SH group and the oxygen of the carbonyl group. What is more interesting, these interactions can lead to the formation of a geometrically advantageous six-membered cyclic ring. And they cause the weakening of the bond between sulfur and hydrogen in the thiol group, which in turn contributes to the high rate of thiol–ene polymerization, when these thiol monomers are used for polymer synthesis. These interactions facilitate the detachment of the hydrogen atom from the SH group, thus facilitating the formation of the thiyl radical. The SH group absorption band of the pure PETMP is located at the wave number 2568.6 cm^−1^ and of pure PETMB at the wavenumber 2563.3 cm^−1^. After addition of TATT ene to the thiols, the shift in the absorption SH bands to the higher wavenumbers is observed, and the $∆\nu_{SH}$ is equal to 2.9 cm^−1^ for a PETMP+TATT mixture and 3.7 cm^−1^ for the PETMB mixture with TATT. It is associated with disruption of SH^.....^SH as well as SH^....^O=C associates. After addition to the thiol–ene system, a solvent mixture of triazolium ionic liquid and propylene carbonate, a further shift in the SH bands toward higher wavenumbers is apparent. Additionally, as can be seen from Figure 4, these shifts are the same regardless of the structure of the ionic liquids, but they are a slightly higher for systems with PETMB thiol.

**Figure 5:**
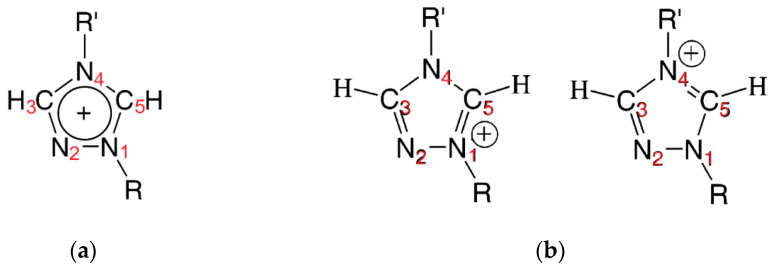
(**a**) Triazole ring of 1,2,4-triazolium ionic liquid cation with number of atoms in the ring; (**b**) resonance structures of 1,2,4-triazolium ionic liquid cation.

Synthesized triazolium ionic liquids have an amphiphilic character, which means that they possess hydrophobic alkyl chains as substituents and a hydrophilic triazole ring. These ionic liquids can be classified on the basis of the Walden plots as “good” ionic liquids, which means that they exhibit poor cation–anion interactions, which are common for ILs with large organic cations, as well as large and/or noncoordinating anions [34]. However, in the case of triazolium ILs, the cation–anion coordination is stronger than in imidazolium ILs due to the increased acidity of the triazolium ring (more acidic C5 hydrogen) [13,34]. The shifts in absorption bands C3-H and C5-H of the triazole ring differ from each other and are higher for the C5-H bands (except ionic liquid with [MMT]^+^ cation). This is due to the more acidic proton of carbon C5 and the structure of the ring which is presented in Figure 5a. As can be seen, the carbon C3 is connected with nitrogen atoms N2 and N4, and the second has an alkyl chain as a substituent. Carbon C5 is connected to nitrogen atoms N1 and N4, and both have an alkyl chain substituent. Furthermore, the resonance structures of this cation (Figure 5b) are the same, and the positive charge is divided equally between nitrogen N1 and N4 [35]. Thus, hydrogen connected to carbon C5 can interact with monomers (their carbonyl group) more strongly than hydrogen connected to the carbon C3. The shifts in these two bands decreased slightly with increasing alkyl chain length of the substituents of studied ILs. As can be seen, for C3-H bands, they are in the range of 2.72–3.64 cm^−1^, and for C5-H, they are in the range 4.44–5.64 cm^−1^ (except [MMT]^+^ cation) and are quite similar for the two types of IL substitution, i.e., 1-methyl-4-alkyl and 1-alkyl-4-methyl, and the two types of thiols used. For the ionic liquid with [MMT]^+^ cation, the same shift is seen for both types of C-H bonds, which indicates that the alkyl chain length of the substituents has an impact on these shifts.

To characterize the used components, the Kamlet–Taft solvatochromic parameters, which described their polarity, were determined. Three parameters can be distinguished: α, which describes the hydrogen bond donation; β, describing hydrogen bonding accepting ability; and π*, the polarizability/dipolarity [27]. These parameters are important for kinetic studies of thiol–ene polymerization performed in the solvent [36], especially, when one of them is an ionic liquid, which is rather a mixture of ions than a simple molecular liquid. The parameters were determined for mixtures of the ionic liquids with propylene carbonate as well as monomers (thiols PETMP and PETMB, and ene TATT). The results obtained are presented in Table 2. The β parameter, which describes hydrogen bond acceptor basicity of the solvent, is independent of the cation structure because it depends mainly on the IL anion structure which is constant in the ionic solvents ([NTf_2_]^−^). The α parameter, hydrogen bond donor acidity, which depends on the cation structure of the ILs, decreases slightly with the increase in the alkyl chain length for both types of the substitution of the 1,2,4-triazole ring. The parameters for the studied thiols vary from the parameters determined for solvents, both α and β parameters, as well as π*. While PETMP has similar β and π* parameters to the solvents and lower α, PETMB differs in all three parameters. It has lower α and π* parameters and, very interestingly, higher β parameters. Thus, PETMB can interact with triazolium cation C-H bonds more strongly than the other thiol PETMP.

### 4.2. Ionogel Synthesis

The focus of this research was on the influence of the structure of the 1,2,4-triazolium ionic liquid, that is, the length of the alkyl of the substituent as well as type of substitution (1-methyl-4-alkyl and 1-alkyl-4-methyl), used in the mixture with propylene carbonate, on the properties of poly(thiol–ene) IG, which can be used as GPE in electrochemical condensers. Thus, it was highly desirable to prepare IG which combines both mechanical strength and good conductive properties. The propylene carbonate allows for higher conductivity but also causes electrolyte leakage from the gel. In the case of the mixture of imidazolium liquid with propylene carbonate, we deal with the leakage problem by modifying it with methacryl-POSS [22]. In the case of triazolium ionic liquids, because of the stronger interactions between components in the system, the ionogels do not leak. Therefore, in these types of materials, the modification of the polymer matrix was not necessary. So, this is the advantage of using triazolium ionic liquid instead of imidazolium ionic liquid.

For the synthesis of ionogels, two types of tetrathiols (PETMP, PETMB) and triene (TATT) were used. The molar ratio of the reactive groups thiol (SH) to ene (C=C) was established to be 1:1. All compositions prior to polymerization were miscible with mixtures of solvents used, but after polymerization, opaque and semi-opaque, quite flexible, and strong ionogels were obtained. The photos gained for the prepared ionogels and the polymer matrix after washing out the solvent from the ionogels are presented in Table 3. The scale of the opacity is added by using +++ for white opaque, ++ for semi-opaque, + for iridescent, and − for fully transparent samples. As can be seen, after removing the solvent from the ionogel’s structure, the opacity of the samples decreases, which indicates phase separation of the components of the prepared ionogels. Our previous research confirms that the photopolymerization reaction between thiol and ene in an ionic liquid occurs as dispersion polymerization [22,26]. This kind of reaction starts with a homogeneous solution of the monomer in an appropriate solvent. However, the polymer is not soluble in it, so the formed polymer precipitates during the polymerization process. Thus, SEM micrographs of prepared ionogels were taken to confirm if that process takes place, and they are collected in Table 3. As can be seen, all the obtained materials consist of microspheres that stick together and differ with the size of the particles, which is in the range of 58 to 189 nm (Table 4). A polymer matrix with smaller particle size was obtained when PETMB thiol was used for ionogel synthesis. Furthermore, the particle size decreased with increasing length of the alkyl substituent in the 1,2,4-triazole ring of IL. The more hydrophobic the ionic liquid is, the smaller the particles are obtained. This may be related to a weakening of the interactions between the ionic liquids and the polymeric matrix. As can be seen in Table 2, when the length of the substituent of the triazole ring substituent increases, from the methyl group to the butyl group, the hydrogen bond donor ability decreases (α parameter). Thus, interactions between the ionic liquid and polymeric matrix decrease, and therefore, the solubility of the polymer in the solvent decreases. This provides earlier nucleation of the polymer during the polymerization process and smaller particles because the amount of the monomers is the same in all of the studied compositions.

Thus, we can conclude that the mixture of IL and PC is a good solvent for used PETMP+TTAT and PETMB+TATT mixtures and a poor solvent for the formation of poly(thiol–ene), which falls out of the solution in the form of microspheres. On the other hand, 1,2,4-triazolium ionic liquids stabilize forming polymer particles and uniformly size particles are obtained. As we stated in our previous work, ionic liquid has an impact on stabilization by formation of solvation shells by hydrogen bonds (between C3-H or/and C5-H bonds of the triazole ring and the C=O groups of the polymer matrix), as well as through electrostatic interactions attracting counterions. The thiol PETMB has a higher ability to accept hydrogen (higher value of β parameter) than PETMP, so it interacts more strongly with ionic liquid, and thus the compatibility of these components is higher. This, in turn, should provide for the acquisition of bigger polymer particles, but the sizes of the obtained polymer particles are smaller. Polymers with a PETMB are more rigid, with higher glass temperature transition (Table 4). This secondary thiol monomer has an additional α-methyl group, which can impede on the rotation of the newly formed thiol–ether bonds (-S-). Probably, this higher stiffness of the material results in the smaller particles of the matrix.

### 4.3. Photopolymerization Kinetics

**Figure 6:**
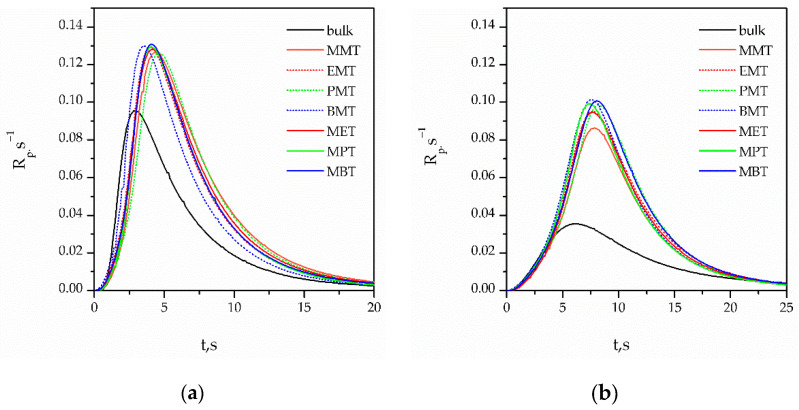
Kinetics curves of photopolymerization of thiol–ene systems (**a**) PETMP+TATT and (**b**) PETMB+TATT in a mixture of 1,4-dialkyl-1,2,4-triazolium ionic liquids with propylene carbonate. Solid line—systems with 1-methyl-4-alkyl-1,2,4-triazolium IL; dashed line—systems with 1-alkyl-4-methyl-1,2,4-triazolium IL.

**Figure 7:**
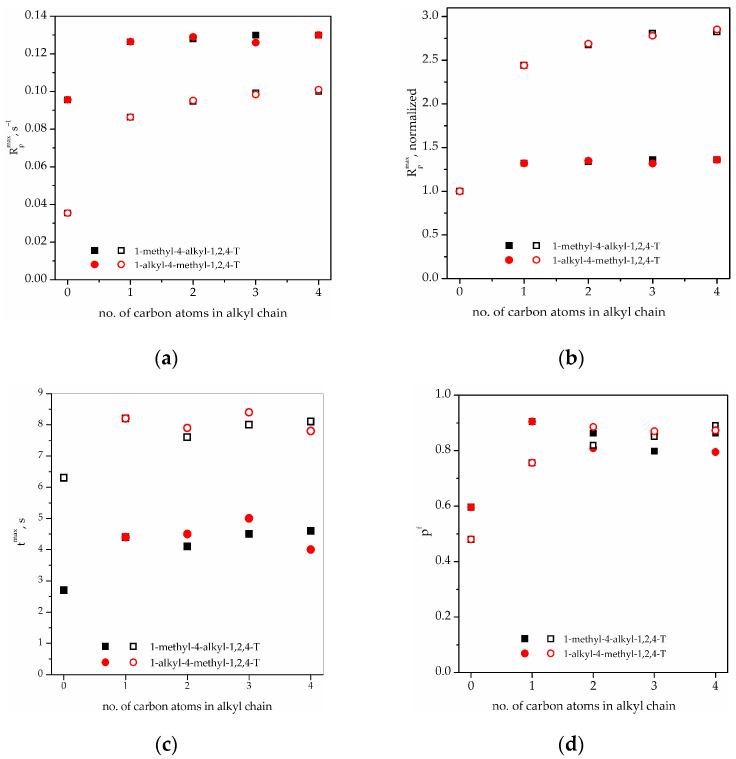
Dependence of (**a**) the maximum polymerization rate, *R_p_^max^*, (**b**) the normalized *R_p_^max^*, (**c**) the time to reach *R_p_^max^*, *t^max^*, and (**d**) the final conversion of functional groups, p^f^ on the length of alkyl chain substituent as well as the type of substitution. Filled symbols—PETMP+TATT system; open symbols—PETMB+TATT system.

The influence of the solvent mixtures of synthesized 1,4-dialkyl-1,2,4-triazolium ionic liquids with propylene carbonate on the photopolymerization kinetics of the investigated systems PETMP+TATT and PETMB+TATT was carried out by photo-differential scanning calorimetry. Thus, the systems studied differ, on one hand, with the use of thiol, i.e., primary thiol PETMP and secondary thiol PETMB, and on the other hand, with the structure of the triazolium cations, that is, the length of the alkyl chain of the substituent and the type of the substitution (1-methyl-4alkyl-1,2,4-triazolium IL and 1-alkyl-4-methyl-1,2,4-triazolium IL). The kinetic curves as a function of the polymerization rate on photopolymerization time (R_p_ = f(t)) are presented in Figure 6. As can be seen, the shapes of the kinetic curves obtained in bulk polymerization, as well as in solution polymerization (in IL/PC mixture), are typical for the thiol–ene reaction. The reaction rate is higher in the system with primary thiol PETMP, and more time to achieve maximum polymerization rate (R_p_^max^) in the system with secondary thiol PETMB is needed. The addition of a solvent mixture to both thiol–ene systems increases the rate of the polymerization process, and there are no substantial differences between the polymerization rates in different ionic liquids used in the study. As we stated earlier, an ionic liquid can cause an increase in the polymerization rate by affecting the stabilization of the transition state which takes place during one of the propagation step reactions, i.e., the chain transfer reaction. Additionally, we can relate the increase in the polymerization rate with the increase in the β Kamlet–Taft parameter [22]. This parameter is related to the anion type of the ionic liquid, and in this study, one type of anion [NTF_2_]^-^ was used. So, the influence of the ILs used is similar. To better show the differences between studied systems (PETMP+TATT and PETMB+TATT) with used ILs, the dependences of the kinetic parameters: R_p_^max^ (as well as normalized R_p_^max^), t^max^ and p^f^ on the length of the alkyl chain substituent, as well as the type of substitution of the ILs, were prepared and are presented in Figure 7. Normalized R_p_^max^ was determined by the deviation of the R_p_^max^ obtained for thiol–ene polymerization in solvent from the R_p_^max^ obtained for bulk polymerization. This allowed to compare the polymerization rate of the two investigated systems in a simple way, as the R_p_^max^ in bulk differed quite a lot. It also allowed to find out what impact the mixture of solvents has on the thiol–ene polymerization of these two systems (Figure 7a,b). There is no influence of the length of the alkyl substituent of 1,2,4-triazolium cation, as well as the type of substitution, on the maximum rate of polymerization of PETMP+TATT system. Thus, the mere addition of the solvent is responsible for the increase in this parameter. In the other system, PETMB+TATT, the type of substitution has no impact on R_p_^max^, too, but the increase in R_p_^max^ with an increasing length of the alkyl substituent is seen. Moreover, the solvents used exerted stronger impact on the polymerization of system with secondary thiol (we can see an increase of 2.5–3 times) than with primary thiol (1.3-fold increase). If the components’ polarities play important roles in the polymerization process, the difference in the Kamlet–Taft β parameter of the used thiols can influence the polymerization process. As can be seen in Table 2, this parameter is higher in the case of PETMB, which can explain the higher increase in R_p_^max^ in this system. Therefore, due to the higher stiffness of the secondary thiol system (additional -CH_3_ group), the final conversion of functional groups in bulk polymerization is lower than in the primary thiol–ene system (Figure 7d). However, in the solvent, the final conversion rises and does not depend on the type of thiol used, nor the type of used IL in the solvent mixture. Thus, the dilution of the systems by the solvent enhances the mobility of the polymerized systems, so the conversion is higher. This is more pronounced in case of the secondary thiol system, which can additionally produce a higher increase in the polymerization rate. Polymerization carried out in solvents needs more time to achieve the maximum polymerization rate (t^max^, Figure 7c), but it does not depend on the type of 1,2,4-triazolium IL.

### 4.4. Ionic Conductivity

**Figure 8:**
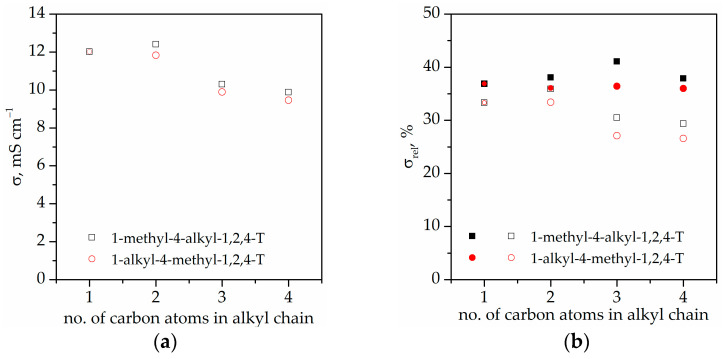
(**a**) Ionic conductivity of the mixtures of ionic liquids with PC. (**b**) Relative conductivity of ionogels. Filled symbols—PETMP+TATT system; open symbols—PETMB+TATT system.

Figure 8 presents the ionic conductivity of the solution of ILs with PC. As can be seen, the ionic conductivity of ILs in PC varies from ca. 9.45 mS·cm^−1^ to ca. 12.40 mS·cm^−1^, depending on the cation structure. Comparing the results of the ionic conductivity of ILs in PC, it could be observed that for IL series with their methyl group substituted in position N1, the ionic conductivity slightly increases from ca. 12.01 mS·cm^−1^ for MMT up to ca. 12.40 mS·cm^−1^ for MET and later decreases to ca. 9.88 mS·cm^−1^ with the increasing alkyl chain length. For IL series with their methyl group substituted in position N4, the ionic conductivity decreases from ca. 12.01 mS·cm^−1^ for MMT to approximately 9.45 mS·cm^−1^ with the increasing alkyl chain length. The decrease in ionic conductivity with the increasing alkyl chain length can be explained by the increasing cation size and their decreasing mobility. It can also be observed that the solution of ILs with the methyl group in position N1 presents slightly higher ionic conductivity than the solution of ILs with the methyl group in position N4. The results clearly show that both the alkyl chain length and its position in the cyclic ring have significant influence on the ionic conductivity of the solutions of ILs in PC.

**Figure 9:**
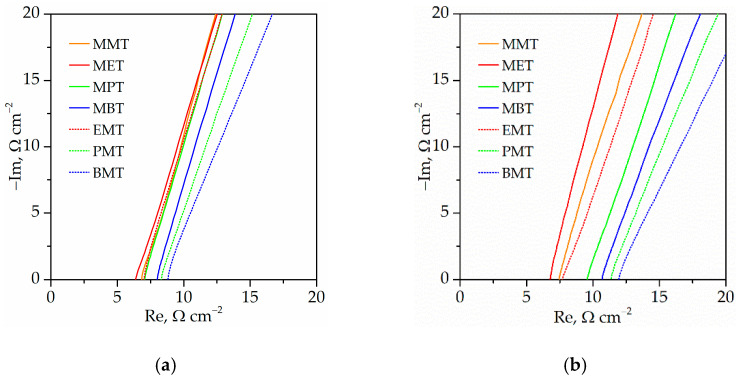
Nyquist plot of gel polymer electrolytes (**a**) gels with PETMP+TATT as polymer matrix (**b**) gels with PETMB+TATT as polymer matrix.

Figure 9 presents a Nyquist plot and Table 5 presents the ionic conductivity of synthesized ionogels with IL solutions in PC. As can be seen, the ionic conductivity varies from ca. 4.7 mS·cm^−1^ to ca. 2.5 mS·cm^−1^. The final ionic conductivity of the ionogels is the result of the initial ionic conductivity of the solution of ILs in PC and the interaction between the electrolyte and the polymer matrix, which is manifested by variable phase separation (Table 3). To eliminate the influence of the ionic conductivity of the electrolyte itself on the ionic conductivity of synthesized ionogels, the relative ionic conductivity (σ_rel_) was calculated. As can be observed, the relative ionic conductivity (Figure 8b) is in the range of 26% to 42%, suggesting that interactions between electrolytes and the polymer matrix play an important role. For TATT+PETMP, as a polymer matrix and series of ionogels with ILs in PC with their methyl group substituted in position N1, the relative ionic conductivity slightly increases from ca. 37% for MMT to 41% for MPT and later decreases to ca. 38% for MBT. For ionogel series containing ILs at PC with their methyl group in position N4, the relative ionic conductivity is similar for all samples and equal to about 36%. The small difference in the relative ionic conductivity of ionogels with TATT+PETMP as polymer matrix is related to their similar phase separation. All ionogels in this series are white and almost nontransparent (Table 3). Different behaviors can be observed for ionogels with TATT+PETMB as the polymer matrix. The ionic conductivity of IGs with the methyl group substituted in position N1 slightly increases from ca. 33% for MMT to 36% for MET and later decrease with increasing alkyl chain length to ca. 29% for MBT. For ionogel series containing ILs with their methyl group at position N4, the ionic conductivity decreases from ca. 33% for MMT to approximately 26% for BMT. All ionogels with TATT+PETMB as a polymer matrix present smaller relative ionic conductivity in comparison with ionogels containing TATT+PETMP. This dependence can be explained by the stronger interactions of the electrolyte with PETMB than with PETMP, which decrease the mobility of the ions. Stronger interactions manifest themselves by smaller phase separation; what is clearly visible for IGs with electrolyte containing MPT/PMT and MBT/BMT is that these samples are partially transparent.

### 4.5. Mechanical Properties

**Figure 10:**
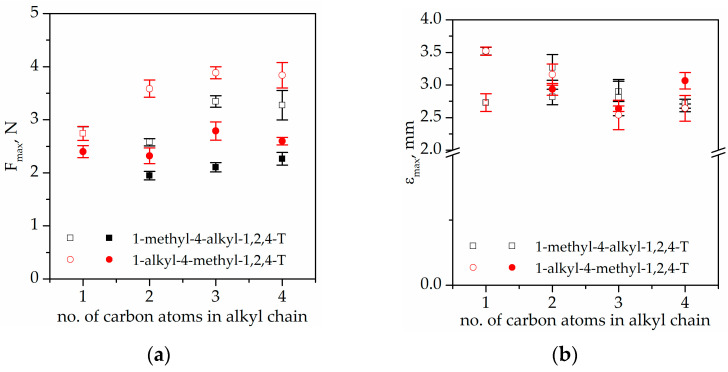
(**a**) Puncture resistance; (**b**) distance at puncture of ionogels. Filled symbols—PETMP+TATT system; open symbols—PETMB+TATT system.

Figure 10 presents the results of the force needed for puncture and elongation at puncture of the prepared IGs. As can be seen, the puncture resistance (Figure 10a) of the ionogels depends on the applied polymer matrix and the structure of the ionic liquids. It can be observed that for TATT+PETMP applied as a polymer matrix, the puncture resistance is equal ca. 2.3–2.4 N for ionogels with MMT and EMT and it later increases to ca. 2.7 N for ionogels containing PMT and BMT, respectively. Different values of puncture resistance are observed for ionogels with methyl group in position N1. The obtained values decreased from ca. 2.4 N for MMT to ca. 1.9 N for MET and later increase to ca. 2.3 N in the series MPT->MBT, which clearly shows that ionogels with ILs containing a methyl group in position N1 present lower puncture resistance than ionogels with ILs containing a methyl group in position N4, especially for shorter alkyl chains. When comparing the elongation at puncture (Figure 10b) of IGs with TATT+PETMP as a polymer matrix, it can be observed that all the elongation values are in the range of ca. 2.7 mm to ca. 3.0 mm, which suggests that there is no significant influence of the structure of triazolium ILs on this parameter. Analyzing the results of the mechanical investigation for ionogels containing TATT+PETMB as a polymer matrix, it can be observed that the resistance to puncture (Figure 10a) is higher in comparison with ionogels containing TATT+PETMP. For ionogels containing poly(TATT+PETMB), the puncture resistance increases with the increasing length of the alkyl chain from ca. 2.7 N for MMT to ca. 3.9 N for PMT/BMT and 3.3 N for MPT/MBT. Ionogels with poly(TATT+PETMB), in a similar way to ionogels with poly(TATT+PETMP), have lower values of puncture resistance when the methyl group is in position N1. When elongation at puncture for ionogels is compared with that of TATT+PETMB as polymer matrix. It can be observed that the elongation decreases with the increasing alkyl chain length from ca. 3.5 mm for MMT to ca. 2.7 mm for PMT/BMT and MPT/MBT. In addition, in the case of ionogels with poly(TATT+PETMB), there is no influence of the position of the methyl group in the structure of ILs on the elongation at puncture. For all synthesized IGs, the mechanical properties, namely, puncture resistance and elongation at puncture, were higher for ionogels with TATT+PETMB than for IGs with TATT+PETMP as a polymer matrix. This observation may be related to the influence of the interactions between the PETMB monomer and the ionic liquids and the influence of these interactions on the process of polymerization and on the structures of the ionogels formed. The occurrence of stronger interactions for ionogels with a matrix containing PETMB was also confirmed by the photos showing phase separation (Table 3), which was also reflected in the values of ionic conductivity.

### 4.6. Investigation of Electrochemical Capacitor

Taking into account the mechanical properties (Figure 10), ionic conductivity (Table 5), and glass transition temperature (Table 4) of synthesized ionogels, it was decided to apply the ionogel with the triazolium ionic liquid with the 1-ethyl-4-methy-1,2,4-triazole cation and with the TATT+PETMP polymer matrix as a gel polymer electrolyte in EDLC. Thanks to its good mechanical properties, it was possible to synthesize polymeric gel with a lower thickness of ca. 200 ± 15 μm.

**Figure 11:**
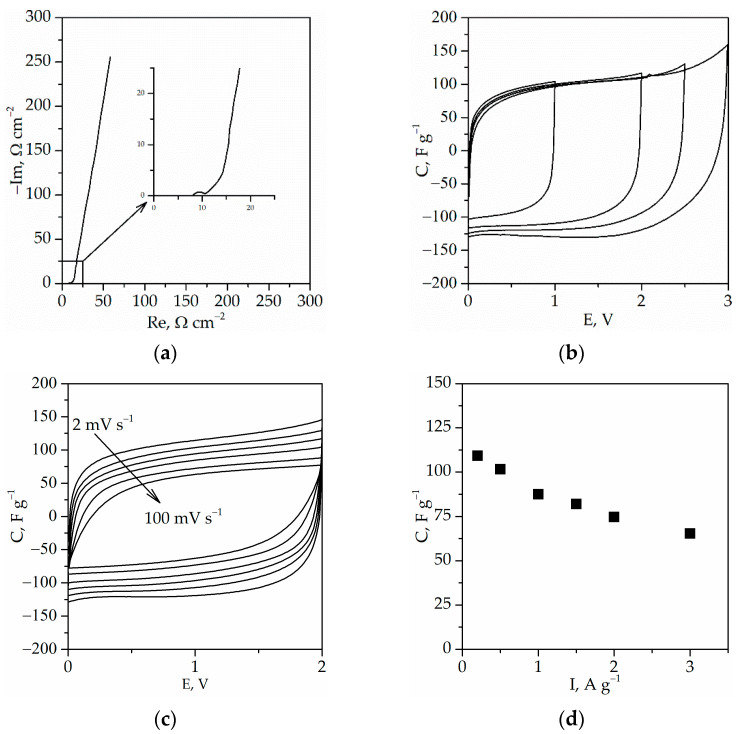
(**a**) Nyquist plot of EDLC; (**b**) cyclic voltammograms up to various values of maximum potential, scan rates 5 mV·s^−1^; (**c**) cyclic voltammograms at scan rates from 2 to 100 mV·s^−1^; (**d**) discharge capacitance vs. current of EDLC. Capacitance and current expressed per AC mass in one electrode.

Figure 11 presents the results of investigation of EDLC with synthesized ionogel. As can be seen from the Nyquist plots (Figure 11a), the equivalent series resistance is equal to ca. 8.1 Ω·cm^2^. This is a slightly higher value in comparison with our previous investigation [22], where ionogels with 1-ethyl-3-methylimidazolium bis(trifluoromethylsulfonyl)imide in PC were used. Moreover, the CV of the electrochemical capacitor is close to box-like in shape, without peaks on the curve, confirming a capacitive behavior without a redox reaction (Figure 11). Additionally, Figure 11c confirms the good properties of EDLC, where, even after increasing the scan rate up to 100 mV·s^−1^, the almost box-like shape of the CV curve can be observed. The good properties of EDLC at faster charging/discharging process are also confirmed by the dependence of the EDLC capacitance on the current (Figure 11d). The obtained results show that the capacitance decreases from ca. 109 F·g^−1^ at 0.2 A·g^−1^ to ca. 65 F·g^−1^ at 3.0 A·g^−1^ with the increasing current, which is about a 40% decrease in capacity. Thus, the obtained gel polymer electrolyte can be applied in EDLC providing good electrochemical properties of the device.

## 5. Conclusions

In summary, triazolium ionic liquids have been successfully synthesized by alkylation of 1,2,4-triazole with 1-bromoalkanes (BE, BP, BB) or iodomethane. Synthesized 1-alkyl-1,2,4-triazoles have an amphiphilic character, which means they possess hydrophobic alkyl chains as substituents and a hydrophilic triazole ring. In the next step, this allowed the synthesis of uniform gel polymer electrolytes by reactions of tetrafunctional thiols with different chemical structures (PETMP, PETMB) and trifunctional ene (TATT) in the presence of a mixture of 1,4-dialkyl-1,2,4-triazolium ionic liquid with PC, in one-pot. For each thiol–ene pair, i.e., PETMB+TATT and PETMP+TATT, in two-component (thiol–ene) and three-component (thiol–ene–solvent) systems, the observed interactions show that in the case of use of triazolium ionic liquids, because of the stronger interactions between the components in the system, the obtained ionogels do not leak. On the basis of the SEM measurements, it can be concluded that the more hydrophobic the ionic liquid is, the smaller the particles of the polymer are obtained. Thus, for the used monomers, the mixture of IL and PC is a very good solvent, but it is a poor solvent for the formation of polymer, which falls out of the solution in the form of microspheres. Nevertheless triazolium ionic liquids stabilize the forming polymer particles, and uniformly sized particles are obtained. Polymers with PETMB are more rigid with a higher glass temperature transition. It can be associated with a hampered rotation of newly created thioether bonds (–S–), which may be caused by another α-methyl group of secondary thiol (PETMB). The influence of the length of the alkyl substituent of 1,2,4-triazolium cation as well as the type of substitution on the maximum rate of the polymerization of the investigated systems can be observed. The presented results clearly show that both the alkyl chain and its position in the cyclic ring has significant influence on the ionic conductivity of the synthesized ILs. For all synthesized ionogels, better mechanical properties—i.e., puncture resistance and elongation at puncture—were obtained for ionogels with TATT+PETMB than TATT+PETMP as the polymer matrix. This observation may be related to the influence of the interactions between the PETMB monomer and the used ionic liquids, and the impact of these interactions on the polymerization process and the structure of the formed ionogels. Good mechanical properties and other significant parameters (i.e., ionic conductivity, glass transition temperature) of the obtained gel polymer electrolyte allowed for the assembly of an electrochemical double layer capacitor with good electrochemical properties of the device.

## Figures and Tables

**Table 1:** Synthesized ionic liquids.

Name	Acronym	Yield, %
1,4-dimethyl-1,2,4-triazolium bis(trifluoromethylsulfonyl)imide	MMT	91.2
4-ethyl-1-methyl-1,2,4-triazolium bis(trifluoromethylsulfonyl)imide	MET	92.7
1-methyl-4-propyl-1,2,4-triazolium bis(trifluoromethylsulfonyl)imide	MPT	93.5
4-butyl-1-methyl-1,2,4-triazolium bis(trifluoromethylsulfonyl)imide	MBT	94.0
1-ethyl-4-methyl-1,2,4-triazolium bis(trifluoromethylsulfonyl)imide	EMT	92.1
4-methyl-1-propyl-1,2,4-triazolium bis(trifluoromethylsulfonyl)imide	PMT	94.1
1-butyl-4-methyl-1,2,4-triazolium bis(trifluoromethylsulfonyl)imide	BMT	86.4

**Table 2:** Solvatochromic parameters (Kamlet–Taft): α—ability to hydrogen bond donation; β—hydrogen bonding accepting ability; π*—the polarizability/dipolarity determined for monomers and mixtures of the synthesized triazolium ionic liquids with PC.

Sample	π*	α	β
TATT	0.63	0.25	0.51
PETMP	0.93	0.49	0.32
PETMB	0.79	0.58	0.40
MMT+PC	0.98	0.78	0.31
MET+PC	0.98	0.76	0.31
EMT+PC	0.98	0.77	0.31
MPT+PC	0.98	0.74	0.31
PMT+PC	0.98	0.75	0.31
MBT+PC	0.98	0.73	0.31
BMT+PC	0.98	0.74	0.31

**Table 3:** Photos of ionogels, as well as photos and SEM micrographs of the polymer matrix after solvent removal for the studied materials with different 1,2,4-triazolium ionic liquids used with propylene carbon as a solvent mixture. The scale of the opacity is as follows: (+++)— white opaque; (++)—semi-opaque, (+)—iridescent, and (−)—fully transparent sample.

Polymer	PETMP+TATT		PETMB+TATT	
IL Cation	Photo	SEM	Photo	SEM
MMT	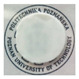 +++ 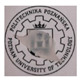 +	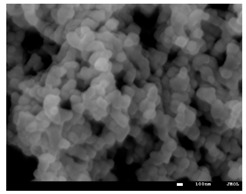	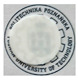 +++ 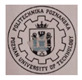 +	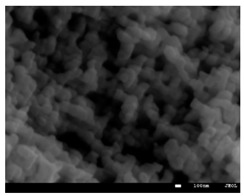
MET	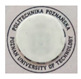 +++ 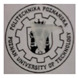 ++	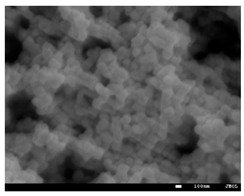	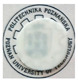 +++ 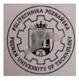 +	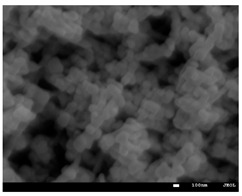
MPT	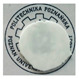 +++ 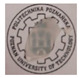 ++	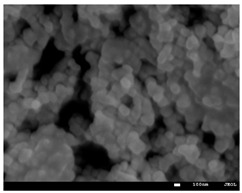	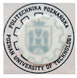 ++ 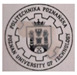 +	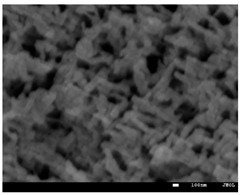
MBT	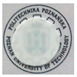 +++ 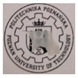 +	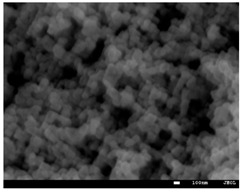	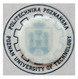 ++ 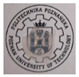 +	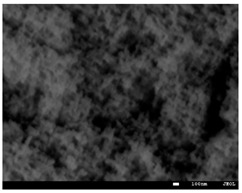
EMT	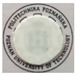 +++ 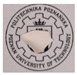 +++	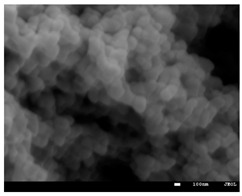	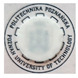 +++ 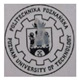 +	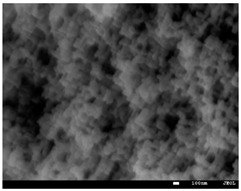
PMT	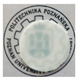 ++ 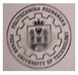 +	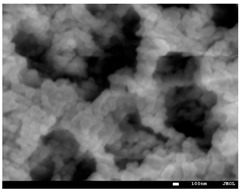	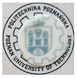 + 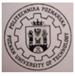 −	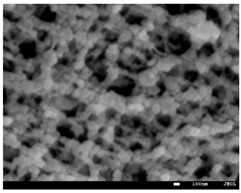
BMT	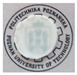 ++ 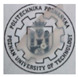 +	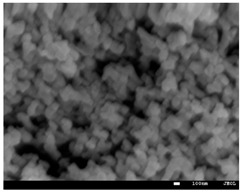	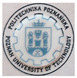 + 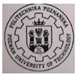 −	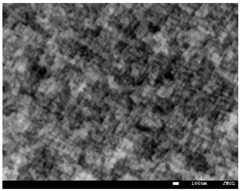

**Table 4:** Size of the polymer particle of the polymer matrix of ionogels (determined from SEM micrographs) and the glass temperature transition Tg of the ionogels.

Polymer Matrix	IL in PC	Particle Size, nm	Tg, °C
TATT+PETMP	-	-	36.6
	MMT	176 ± 37	10.3
	EMT	189 ± 44	7.6
	MET	160 ± 20	10.0
	PMT	152 ± 24	9.6
	MPT	155 ± 26	8.6
	BMT	146 ± 22	6.6
	MBT	122 ± 22	8.3
TATT+PETMB	-	-	34.1
	MMT	152 ± 25	18.6
	EMT	91 ± 15	17.6
	MET	123 ± 19	19.3
	PMT	99 ± 17	19.0
	MPT	98 ± 19	21.32
	BMT	64 ± 10	18.00
	MBT	58 ± 8	19.96

**Table 5:** Ionic conductivity of IGs with the mixture of IL with PC as electrolyte and different polymer matrix.

IL in PC	Conductivity, mS·cm^−1^	
	TATT+PETMP	TATT+PETMB
MMT	4.43 ± 0.08	4.02 ± 0.11
MET	4.72 ± 0.15	4.45 ± 0.09
MPT	4.23 ± 0.13	3.14 ± 0.014
MBT	3.74 ± 0.09	2.9 ± 0.11
EMT	4.27 ± 0.06	3.95 ± 0.12
PMT	3.60 ± 0.11	2.68 ± 0.13
BMT	3.4 ± 0.07	2.51 ± 0.10

## Data Availability

Not applicable.
